# Olive oil-derived monounsaturated fat influences metabolic signatures in serotonergic regions of the brain in broiler chicken

**DOI:** 10.3389/fphys.2026.1826795

**Published:** 2026-05-21

**Authors:** Odinaka C. Iwuozo, Paul C. Omaliko, Nathanael I. Lichti, Bruce R. Cooper, Yewande O. Fasina

**Affiliations:** 1 Department of Animal Sciences, North Carolina A&T State University, Greensboro, NC, United States; 2 Bindley Bioscience Center, Purdue University, West Lafayette, IN, United States

**Keywords:** broiler chickens, dorsal raphe nucleus, hypothalamus, metabolomics, olive oil

## Abstract

**Introduction:**

Dietary fat modulates the dorsal raphe nucleus (DRN) and hypothalamic (HYP) serotonergic regions of the brain, influencing neurotransmitter activity and behavior in broiler chickens. Identifying metabolites that reach these serotonergic regions in response to dietary fat may support the formulation of diets that enhance neurological function and improve wellbeing. This study aimed to determine how monounsaturated fatty acids (MUFA) from olive oil influence the composition of metabolites that reach the DRN and HYP in broiler chickens.

**Methods:**

Day-old Ross 708 chicks (n = 160) were weighed and randomly assigned to two dietary treatments consisting of a corn-soybean meal basal diet supplemented with either poultry fat (CON) or olive oil (OLIV) at 3%, with five replicate pens of 16 chicks each. On d 20, plasma, DRN and HYP were aseptically collected and subjected to HPLCMS analysis for serotonin and metabolite profiling. Serotonin concentrations were analyzed using a Student’s *t*-test, and metabolomic data were evaluated using linear modeling and principal component analysis to identify distinct metabolite profiles. Differentially abundant metabolites were annotated using HMDB and KEGG databases, followed by pathway enrichment analysis.

**Results and discussion:**

Results showed higher (*P* < 0.05) serotonin concentrations in both the DRN and HYP of OLIVfed birds. A total of 15,084 metabolites were differentially abundant (*P* < 0.05), of which 617 were annotated. Among these, Ssuccinylcysteine was upregulated in both plasma and DRN, while sphingomyelin was upregulated in DRN but downregulated in plasma. Dimethoxyflavone and cytosine were upregulated in HYP. Gammaglutamylleucine and epigallocatechin metabolites were differentially abundant in plasma and HYP but downregulated in DRN. Enrichment analysis revealed that glutamate metabolism was enriched in plasma and HYP but not significantly in DRN, whereas aspartate metabolism was altered in plasma and DRN only. Metabolic changes associated with purine metabolism across plasma, DRN, and HYP, and alterations in alanine, aspartate, and glutamate metabolism were also noted. Overall, dietary olive oil altered metabolites and pathways in serotonergic brain regions, indicating that MUFA intake modulates their metabolic activity. Higher serotonin concentrations in the DRN and HYP further support enhanced central serotonergic function in OLIV fed broiler chickens.

## 1 Introduction

Dietary fat composition plays a critical role in modulating brain function and overall physiological health, influencing neural signaling, plasticity, and behavior (Chianese et al., 2018). This dietary fat permeates serotonergic brain regions such as the dorsal raphe nucleus (DRN) and hypothalamus (HYP) to influence physiological and behavioral processes (Gray et al., 2026). These regions are critical for regulating mood, appetite, and stress responses, mainly through their modulation of serotonergic signaling, a key neurotransmitter system involved in emotional and behavioral regulation (Patrick and Ames, 2015). DRN is a primary source of serotonin in the brain and is particularly vulnerable to dietary fat-induced changes (Aklan et al., 2023). Serotonergic signaling in the DRN is known to regulate mood and emotional behavior, and alterations in this system have been linked to disorders such as oxidative damage, depression and anxiety in rodents (Albert et al., 2014; Nishitani et al., 2019). Recent studies have demonstrated that diets high in saturated fatty acids (SFAs) such as poultry fat can impair serotonergic function by promoting neuroinflammation and oxidative stress (Tan and Norhaizan, 2019), whereas polyunsaturated fatty acids (PUFAs), particularly omega-3 fatty acids, exert protective effects by enhancing synaptic plasticity and reducing inflammatory markers within serotonergic nuclei such as the DRN (Larrieu and Layé, 2018). Similarly, HYP is a key regulator of energy homeostasis, thermoregulation and feeding behavior, profoundly influenced by dietary fats. The HYP neurons integrate peripheral signals to modulate appetite and energy expenditure, and their function is sensitive to the lipid composition of the diet (Valdearcos et al., 2014). High-fat diets rich in SFAs have been shown to induce hypothalamic inflammation and leptin resistance, contributing to dysregulated feeding behavior and obesity (Thaler et al., 2012). In contrast, diets enriched with PUFAs such as omega-3 fatty acids have been associated with improved hypothalamic function and reduced inflammation, highlighting the potential of specific dietary fats to mitigate metabolic and behavioral dysregulation (Cintra et al., 2012).

The brain is highly susceptible to oxidative stress and inflammation, which are key contributors to neurodegenerative diseases and mood disorders (Hassamal, 2023). Monounsaturated fatty acids (MUFAs) such as olive oil, rich in oleic acid, has garnered significant attention for its potential neuroprotective and behavioral benefits. MUFAs are known to play a crucial role in maintaining cell membrane integrity, modulating inflammation, and supporting synaptic plasticity, all of which are essential for optimal brain function (Riviere et al., 2021). Along with its polyphenolic compounds, olive oil’s high MUFAs content has been shown to exert antioxidant and anti-inflammatory effects, which can particularly be beneficial for brain health (Martín‐Peláez et al., 2013; Angeloni et al., 2017). The polyphenols have been associated with numerous health benefits, including improved lipid profiles, enhanced antioxidant status, modulation of inflammatory responses, and improved feed efficiency in poultry (Pirman et al., 2021; Rafei-Tari et al., 2021). Specifically, olive oil’s polyphenols, such as hydroxytyrosol and oleocanthal, have been demonstrated to reduce oxidative damage and inhibit pro-inflammatory pathways in the human brain (Pérez-Herrera et al., 2012). Additionally, olive oil has been linked to improved memory and learning in animal models, suggesting its potential to support neurogenesis and synaptic plasticity in broiler chickens (Lauretti et al., 2017). These properties make olive oil a promising dietary intervention for mitigating cognitive decline and improving mood and behavior in humans (Román et al., 2019), which also is envisaged to have such potentials in broiler chickens.

The brain is a metabolically active organ relying on the efficient transport of essential nutrients and molecules to maintain neural function, cognitive development, and stress resilience in poultry (Tonissen et al., 2022). However, the influence of MUFAs on the composition of metabolites that reach serotonergic regions of the brain, and their potential to drive metabolic or behavioral changes remain largely unexplored in broiler chickens. This study employs a metabolomic approach to investigate the influence of MUFAs in the form of olive oil on the composition of metabolites and biochemical pathways in plasma and in the serotonin-rich regions of the brain, namely DRN and HYP. By examining these metabolomic shifts, we seek to explore the mechanisms by which different dietary fats influence DRN, HYP, and serotonergic signaling, providing comprehensive understanding of the role of dietary fat in brain health, thus informing dietary strategies for the prevention and management of neurobehavioral disorders in poultry.

## 2 Materials and methods

### 2.1 Animals, housing and diet composition

Day-old (Ross 708) broiler male chicks (n =160) were commercially sourced and housed at the Poultry Research Unit of the North Carolina A&T State University (Greensboro, NC) in a 3-weeks study. Chicks were weighed and randomly assigned to two treatment groups, each treatment group was randomly allocated to 5 replicate pens, containing 16 chicks each, and allowed free access to water throughout the experiment. Growth performance parameters such as body weight (BW), feed intake (FI), body weight gain (BWG) and feed conversion ratio (FCR) were determined on d 21. The chicks were housed in battery cages (Alternative Design Manufacturing and amp Supply Inc., Siloam Springs, AR) from d 1 to d 21 of the study. Each battery cage had a nipple drinker to supply water and a feeder tray which was adjusted in height for reach according to the progressive growth of the chicks. The bird housing was set at a temperature of 33 °C from d 1 to d 7, and 31 °C from d 8 to d 21. Photoperiod consisted of continuous (23L:1D) lighting at 30 lux from placement to 21 d (Omaliko et al., 2024). The treatments included a basal diet of corn-soybean meal (SBM) with 3% dietary fat inclusions as either poultry fat (CON, mainly SFAs) or olive oil (OLIV, primarily MUFAs), as presented in **Table 1**. The experimental diets were produced at the North Carolina State University Feed Education Unit (Raleigh, NC) and calculated to be isocaloric. The fat types were procured commercially from Jedwards International, Inc. (Braintree, MA). The experimental diets of starter pellet crumbles (**Table 1**) were provided *ad libitum* to the chicks throughout the study, formulated to meet or slightly exceed nutritional requirements following guidelines outlined in the Ross broiler nutrition specification handbook (Aviagen, W, 2022). The fatty acid compositions of the experimental diets are presented in **Table 2**.

**Table 1:** Composition of experimental starter diets (% “as is”)^1^.

Ingredients	CON	OLIV
Corn	53.22	53.22
Soybean Meal	39.40	39.40
Fat/Oil*	3.00	3.00
Mono-Dicalcium Phosphate	1.81	1.81
Limestone 37%	0.95	0.95
Salt NaCl	0.45	0.45
DL-Methionine	0.35	0.35
NCSU Poultry Mineral Premix^2^	0.20	0.20
Choline Chloride 60%	0.20	0.20
L-Lysine	0.18	0.18
L-Threonine	0.09	0.09
NCSU Poultry Vitamin Premix^3^	0.05	0.05
Selenium Premix^+^	0.05	0.05
Santoquin	0.05	0.05
Analyzed nutrient composition^4^		
Metabolizable Energy (Kcal/kg)	3,152.6	3,106.4
Crude Protein, %	23.06	24.38
Crude Fat, %	5.42	5.18
Crude Fiber, %	2.1	2.3
Ash, %	5.64	5.73
Calculated nutrient composition		
Total Sulfur Amino Acids, %	0.19	0.19
Lysine, %	1.44	1.44
Calcium, %	0.96	0.96
Available phosphorus, %	0.48	0.48

^1^ Diets used in the study included the following: i) conventional Corn-soybean meal with the addition of poultry fat (SFAs) as (CON diet); ii) conventional Corn-SBM in which olive oil (MUFAs) was incorporated as (OLIV). Each of these 2 diets were separately formulated for the starter (d 1 to 21) of study.

*2 different fat types were added at 3% in each diet.

^2^ Mineral Premix, supplied per kilogram of diet: Manganese (Mn), 60 mg; Zinc (Zn), 60 mg; Iron (Fe), 40 mg; Copper (Cu), 5 mg; Iodine (I), 1.2mg; Cobalt (Co), 0.5 mg.

^3^ Vitamin Premix, supplied per kilogram of diet: Vitamin A (6,600 IU), Vitamin D (1,980 IU), Vitamin E (33 IU), Vitamin B12 (0.02 mg), Biotin (0.13 mg), Menadione (1.98 mg), Thiamine (1.98 mg), Riboflavin (6.60 mg), d-Pantothenic Acid (11.0 mg), Vitamin B6 (3.96 mg), Niacin (55.0 mg), Folic Acid (1.1 mg).

^4^ Diets were analyzed for proximate nutrient composition by Eurofins Scientific Inc. Nutrient Analysis Center, 2200 Rittenhouse Street, Suite 150, Des Moines, IA 50321.

^+^ Selenium Premix provides 0.3 mg Selenium/Kg of feed as sodium selenite.

**Table 2:** Experimental diets fatty acid composition^1^.

Starter diets		
Fatty acids	CON (%)	OLIV (%)
C4:0	< 0.02	< 0.02
C6:0	< 0.02	< 0.02
C8:0	< 0.02	< 0.02
C10:0	< 0.02	< 0.02
C11:0	< 0.02	< 0.02
C12:0	< 0.02	< 0.02
C14:0	0.02	< 0.02
C14:1	< 0.02	< 0.02
C15:0	< 0.02	< 0.02
C15:1	< 0.02	< 0.02
C16:0	1.12	0.74
C16:1	0.19	0.05
C16:2	< 0.02	< 0.02
C16:3	< 0.02	< 0.02
C16:4	< 0.02	< 0.02
C17:0	< 0.02	< 0.02
C17:1	< 0.02	< 0.02
C18:0	0.25	0.16
C18:1	1.75	2.39
C18:2	1.95	1.58
C18:3 n-3	0.12	0.11
C18:3 n-6	< 0.02	< 0.02
C18:4	< 0.02	< 0.02
C20:0	< 0.02	< 0.02
C20:1	< 0.02	< 0.02
C20:2	< 0.02	< 0.02
C20:3 n-3	< 0.02	< 0.02
C20:3 n-6	< 0.02	< 0.02
C20:4 n-3	< 0.02	< 0.02
C20:4 n-6	< 0.02	< 0.02
C20:5 n-3	< 0.02	< 0.02
C21:5	< 0.02	< 0.02
C22:0	< 0.02	< 0.02
C22:1	< 0.02	< 0.02
C22:2	< 0.02	< 0.02
C22:3	< 0.02	< 0.02
C22:4	< 0.02	< 0.02
C22:5 n-3	< 0.02	< 0.02
C22:5 n-6	< 0.02	< 0.02
C22:6	< 0.02	< 0.02
C24:0	< 0.02	< 0.02
C24:1	< 0.02	< 0.02
Total n3	0.12	0.11
Total n5	< 0.05	< 0.05
Total n6	1.99	1.59
Total n7	0.27	0.13
Total n9	1.77	2.41
Total fatty acids	5.67	5.23
MUFAs	2.07	2.56
PUFAs	2.13	1.71
SFAs	1.44	0.96

^1^ The fatty acid composition of the diets were analyzed by Eurofins Scientific Inc. Nutrient Analysis Center, 2200 Rittenhouse Street, Suite 150, Des Moines, IA 50321.

PUFA, polyunsaturated fatty acids**;** MUFA, monounsaturated fatty acids; SFA, Saturated fatty acids.

### 2.2 Sample collection

On d 20, one bird was randomly taken from each pen and weight recorded prior to sampling, totaling 5 birds per treatment. Blood was collected from the brachial (wing) vein using a sterile 23 gauge 1″ needle attached to prelabeled sterile Ethylenediaminetetraacetic acid (EDTA) vacutainer tubes. Thereafter, the blood samples were centrifuged at 1,500 × *g* for 10 min to recover platelet-free plasma. The plasma was carefully collected and transferred into 1.5 mL Eppendorf tubes. The birds were euthanized using carbon dioxide (CO_2_) asphyxiation exposure in accordance with the institution’s Institutional Animal Care and Use Committee (IACUC) approved protocol (IACUC 20-004.0), which specifies an inhaled concentration of 80% CO_2_. The head was cut off, and thereafter brain tissues (DRN and HYP) were aseptically collected on ice and immediately placed into 2.0 mL cryogenic tube, quickly frozen in liquid nitrogen. Both plasma and brain tissues were subsequently stored at -80 °C until time of use.

### 2.3 Determination of serotonin concentration using LC–MS/MS

An Agilent 1290 Infinity II liquid chromatography (LC) system coupled to an Agilent 6470 series QQQ mass spectrometer (MS/MS) was used to analyze samples. (Agilent Technologies, Santa Clara, CA). An Acquity UPLC BEH Amide 2.1 mm x 100 mm, 1.7 µm column was used for LC separation (Waters Corp. Milford, MA). The buffers were A) acetonitrile + 0.3% formic acid and B) acetonitrile/100 mM ammonium formate (20/80 v/v). The linear LC gradient was as follows: time 0 minutes, 0% B; time 1 minutes, 0% B; time 10 minutes, 50% B; time 11 minutes, 100% B; time 11.5 minutes, 0% B; time 15 minutes, 0% B. The flow rate was 0.3 mL/min and the column was heated to 30 °C. Multiple reaction monitoring was used for MS analysis. Data were acquired in positive electrospray ionization (ESI) mode. The jet stream ESI interface had a gas temperature of 325 °C, gas flow rate of 8 L/minute, nebulizer pressure of 40 psi, sheath gas temperature of 250 °C, sheath gas flow rate of 7 L/minute, capillary voltage of 4000 V in positive mode, and nozzle voltage of 1500 V. The ΔEMV voltage was 500 V. Agilent Masshunter Quantitative analysis software was used for data analysis (version 10.1).

### 2.4 Preparation of sample for LC-MS untargeted metabolomic

Frozen plasma, DRN and HYP samples were partially thawed on ice; 100 µL plasma was pipetted into a microcentrifuge tube and 100 mg brain tissue was weighed from each sample into a CK14 Precelly tubes (precellys 24 bertin technologies, Rockville MD USA) and homogenized at 2500 rpm for 15 s. Thereafter, 1 mL of 80% methanol extraction solvent was added to the plasma and brain tissue homogenates and re-homogenized. Subsequently, the liquid was transferred into fresh microcentrifuge tubes and then centrifuged (Sorvall Legend Micro 21, Thermo Scientific, Waltham, MA USA) for 8 min at 10, 000 rpm. The supernatant after transfer to new microcentrifuge tube was evaporated in a Speedvac (SAVANT SPD2010, Thermo Scientific, Waltham, MA USA) overnight. The aftermath dried extract was then reconstituted for HPLC-MS analysis by adding 75 uL of 95% water, 5% acetonitrile, 0.1% formic acid. The tubes were sonicated for 5 minutes and centrifuged for 8 min at 13, 000 rpm. Finally, the supernatants were transferred to a Waters plastic HPLC vial (Waters Corp. Milford, MA 01757) and the pellet discarded. Pooled quality control (QC) samples were prepared by combining aliquots from all the samples and were analyzed periodically during the analytic run to monitor the performance of the system.

### 2.5 LC-MS untargeted metabolomic analysis

An Agilent 1290 Infinity II liquid chromatography system was coupled to an Agilent 6546 series Q-TOF mass spectrometer (Agilent Technologies, Santa Clara, CA). An Atlantis T3 2.1 mm x 150 mm, 3.0 µm column was used for LC separation (Waters Corp. Milford, MA). The buffers were A) water + 0.1% formic acid and B) acetonitrile + 0.1% formic acid. The linear LC gradient was as follows: time 0 min, 0% B; time 1.5 min, 0% B; time 24 min, 20% B; time 31.5 min, 95% B; time 33.8 min, 95% B; time 35.3 min, 0% B, and time 42.8 min, 0% B. The flow rate was 0.3 mL/min, and the column was heated to 40 °C. Data were acquired in positive electrospray ionization (ESI) mode. The jet stream ESI interface had a gas temperature of 325 °C, gas flow rate of 8 L/min, nebulizer pressure of 30 psi, sheath gas temperature of 300 °C, sheath gas flow rate of 7 L/min, capillary voltage of 3500 V. The injection volume for each sample was 4 μL. MS/MS was performed in Data Dependent Analysis (DDA) mode, with a range of 70–1000 m/z for MS (5 Hz) and 40–1000 for MS/MS (3 Hz). Fixed collision energies of 10, 20, and 40 eV were used. Mass accuracy was ensured by infusing Agilent Reference Mass Correction Solution (G1969-85001). Furthermore, to evaluate the stability of the LC-MS during the whole acquisition, a quality control sample (pool of all samples) was acquired after every 10 samples.

### 2.6 Data processing of the metabolites

The acquired MS data pretreatments include peak detection, peak grouping, alignment and gap filling for m/z values, retention time correction, second peak grouping, and annotation of isotopes and adducts were performed using MS-DIAL software (v. 4.9) (Tsugawa et al, 2015). The datasets were transformed then normalized prior to analysis. Each unique ion was identified by its corresponding retention time (RT) and *m*/*z* value. Intensities of each peak were recorded and a matrix containing assigned peak indices (retention time-*m*/*z* pairs), sample names (observations), and ion intensity information (variables) was generated. The intensity of peak data was further pre-processed by MS-DIAL, the features that were detected in less than 60% of QC samples were removed, the remaining peaks with missing values were imputed with the k-nearest neighbor algorithm to further improve the data quality. PCA was performed for outlier detection and batch effects evaluation using the pre-processed dataset. Quality control-based signal correction was fitted to the QC data with respect to the order of injections to minimize signal intensity drift over time. The peaks in the mzXML in which the centWave algorithm was used for high-resolution centroid data feature detection, ppm = 20 for the TOF instrument; The grouping method for peak alignment in mzClust was used for high resolution alignment, mzppm (the relative error used for clustering) = 30 ppm, minsamp (the minimum number of samples in one bin) = 1, minfrac (the minimum fraction of each class in one bin) = 0.5; retention time correction was performed by rector-methods using the obiwarp algorithm; re-alignment was performed using the group method with tighter range; missing peaks were solved using the fill Peaks method; and data in CSV files were exported for further analyses. Multivariate analysis techniques were then used to determine the differences within each sample (Worley and Powers, 2013). For MS1 identification, the adducts [M+H]^+^, [M+Na]^+^, [2M+H]^+^, and [2M + Na]^+^ were used in the positive mode, with 0.01 Da mass tolerance. For MS2 annotation, mass tolerance was set at 0.05 Da with an identification score cut-off of 75%. Open-source Kyoto Encyclopedia of Genes and Genomes (KEGG) and Human Metabolome Database (HMDB) databases were used to annotate the metabolites by matching the exact molecular mass data (*m*/*z*) of samples with those from the database. If a mass difference between observed and the database value was less than 10 ppm, the metabolite would be annotated, and the molecular formula of metabolites would further be identified and validated by the isotopic distribution measurements.

### 2.7 Statistical analysis

Growth performance parameters and serotonin concentration data were analyzed using a Student’s *t*-test to assess differences between the CON and OLIV treatments. For untargeted metabolomics data, a multivariate analysis was performed using Principal Component Analysis (PCA) to reduce data dimensionality and to visualize clustering patterns and separations among samples based on their metabolite profiles. The PCA was generated using only metabolites that exhibited significant differences between treatment groups for each tissue. Also, a univariate linear model with fixed effects of treatment and tissue was fit to each peak and conditional contrasts were used to identify metabolites that were significantly different between the two treatments within each sample. Metabolites detected in plasma and concurrently present in either the DRN or HYP were considered candidates for systemic migration into serotonergic brain regions. The statistical analysis was performed using R v4.4.1 software (R Core Team, 2024) equipped with the emmeans (Lenth, 2024)) and mixOmics (Rohart et al., 2017) packages, as well as in-house packages for workflow management. To elucidate the distinct metabolic pathways in the plasma and brain regions, MetaboAnalyst 6.0 (https://www.metaboanalyst.ca/) open access software was used for pathway enrichment analysis and pathway topology analysis, employing KEGG pathway database (Kanehisa and Goto, 2000), and *Gallus gallus* library of KEGG, respectively to identify metabolic pathway profile changes in the plasma and brain segments. The enriched and metabolomic topology pathways were considered at -log10 (*P*) > 1.3 (equivalent of *P* < 0.05) and pathway impact > 0.05, respectively.

## 3 Results

### 3.1 Number of metabolites detected in a dataset and principal component analysis

**Figure 1:**
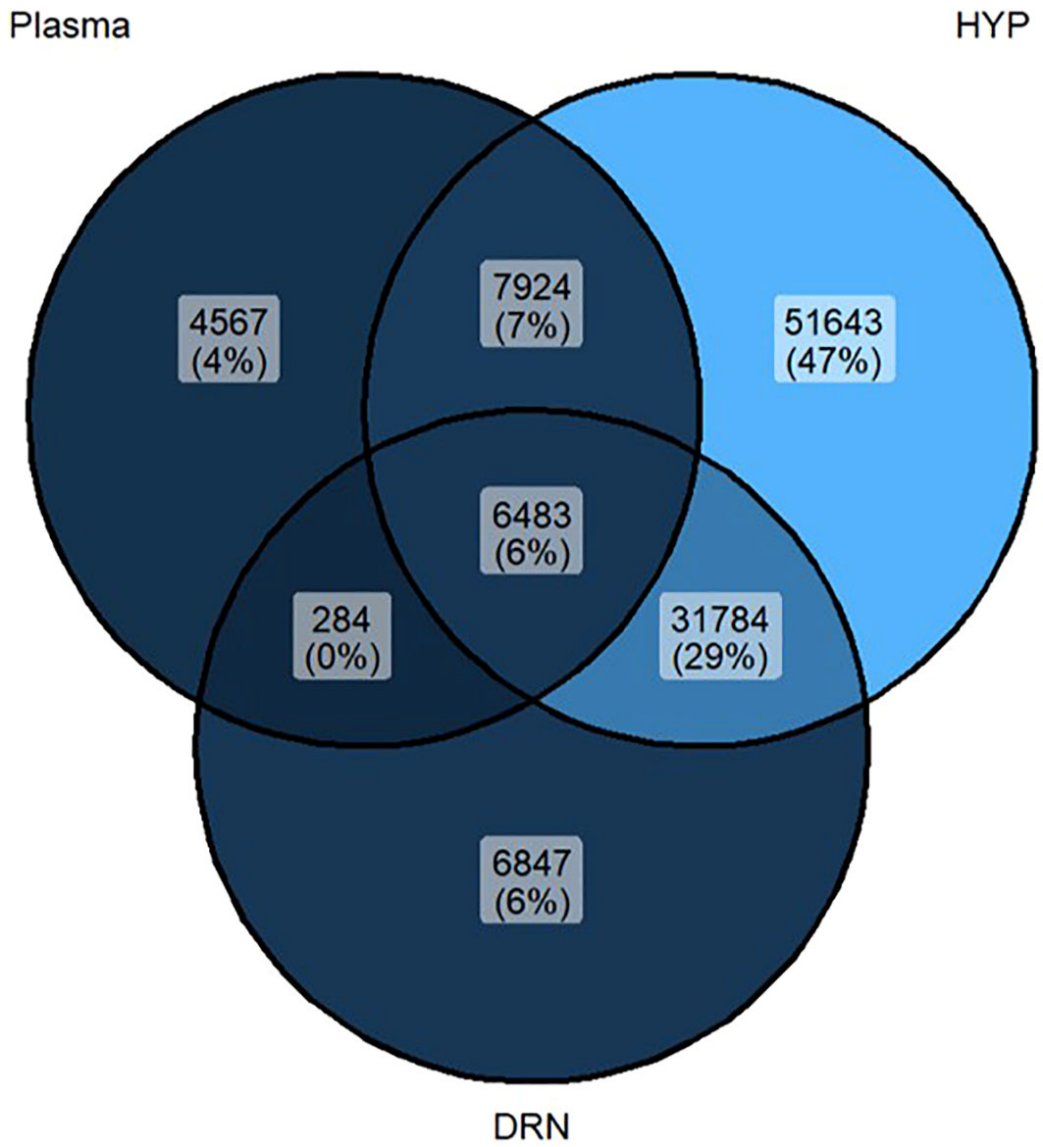
Number of metabolites that pass missing value and blank filters for OLIV.

**Figure 2:**
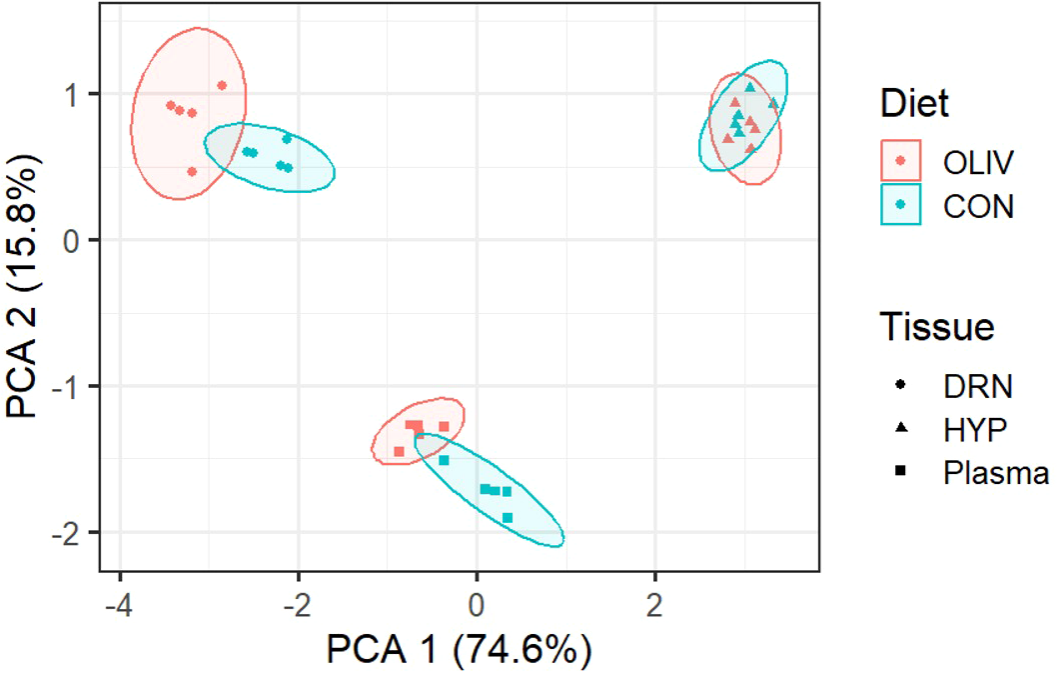
PCA score plot showing distribution of differential metabolites in plasma, DRN and HYP.

The results showed that birds had similar (*P >* 0.05) growth performances such as BW, FI, BWG and FCR as presented in **Table 3**. In this study, LC-MS untargeted metabolomics was employed to analyze the metabolite profiles contrast between CON and OLIV samples obtained from three distinct samples namely, plasma, DRN and HYP. Data analysis showed a total of 109,532 detected metabolites, of which the number of shared (17%, 41% and 89%) and unique (6%, 4% and 47%) metabolites detected in the dataset in the plasma, DRN and HYP, respectively that pass the missing value and blank filter for OLIV are shown in the Venn diagram (**Figure 1**). However, only 15,084 metabolites were differentially abundant (*P <* 0.05) of which 617 metabolites were annotated. The PCA score plot of CON and OLIV groups are presented in **Figure 2**. The PCA demonstrated a distinct clustering of CON and OLIV groups in plasma and DRN, whereas HYP exhibited partial overlap.

**Table 3:** Effect of dietary fat on growth performance of broiler chicken (d 1–21).

Treatments	BW (kg/bird)	FI (kg/bird)	BWG (kg/bird)	FCR (kg/kg)
CON	0.859	0.966	0.838	1.188
OLIV	0.880	0.996	0.813	1.188
*P* -value	0.113	0.069	0.099	1.000

### 3.2 Serotonin profile by LC–MS/MS analysis

Serotonin concentrations in plasma, DRN, and HYP of the broiler chickens in the CON and OLIV treatments are presented in **Table 4**. Plasma serotonin levels were higher in the OLIV group, approaching significance (*P* = 0.0622). In the serotoninrich brain regions, both the DRN and HYP also exhibited higher (*P* < 0.05) serotonin concentrations in OLIV compared with CON.

**Table 4:** Serotonin concentration in broiler chickens fed dietary fat.

Treatments	Plasma (ng/mg tissue)	DRN (ng/mg tissue)	HYP (ng/mg tissue)
CON	0.5083	0.0117	0.0409
OLIV	2.1390	0.0255	0.0481
*P*-value	0.0622	0.0032	0.0007

### 3.3 Differentially abundant metabolites in plasma and brain regions

Metabolites abundant in plasma and detected in the DRN and/or HYP were interpreted as putative circulating metabolites with potential access to serotonergic brain regions. The differential metabolites that are abundant in plasma and DRN or HYP, or both are presented in **Table 5**. Among these metabolites, 3 were specifically abundant in plasma and DRN only, including S-succinylcysteine upregulated (*P <* 0.05) in both plasma and DRN, and sphingomyelin d18:1-C20:0 upregulated in DRN but downregulated in plasma. Also, Ala-Val was downregulated (*P <* 0.05) in both plasma and DRN. Similarly, 3 metabolites were differentially abundant in plasma and HYP only, such as dimethoxyflavone upregulated (*P <* 0.05) in HYP, but downregulated in plasma, while cytosine and santin were downregulated in both plasma and HYP. Additionally, 3 metabolites were differentially abundant in plasma, DRN and HYP. These include γ-glutamylleucine, epigallocatechin, and dethiobiotin either up- or downregulated, or both in these tissues.

**Table 5:** Differentially abundant metabolites in plasma and brain segments.

Metabolite name	Average Mz	*P*-value	Plasma	DRN	HYP	Putative functions
S-succinylcysteine	238.040	0.034	Up	Up	–	Amplify serotonergic signaling to enhance neuroprotection and reduced aggression (Jové et al., 2020)
Sphingomyelin d18:1-C20:0	743.324	0.003	Down	Up	–	Enhance myelination and neural function (Schneider et al., 2019)
Ala-Val	187.108	0.027	Down	Down	–	Reduce neuroinflammation and maintain brain functions (Küpeli Akkol et al., 2020; Kornicka et al., 2023)
Dimethoxyflavone	265.085	0.012	Down	–	Up	Inhibit butyrylcholinesterase regulating neurotransmitter activity (Tomou et al., 2020)
Cytosine	112.051	0.036	Down	–	Down	Induce epigenetic modifications (Li et al., 2015)
Santin	343.108	0.025	Down	–	Down	Enhance memory and cognitive capacity (Li et al., 2022)
γ -Glutamylleucine	261.145	0.001	Up	Down	Up	Modulates GABAergic signaling by reducing stress-induced hyperactivity (Wu et al., 2022)
Epigallocatechin	307.083	0.023	Up	Down	Up	Normalize hypothalamic-pituitary-adrenal axis activity to improve stress (Chi et al., 2020)
Dethiobiotin	213.124	0.010	Down	Down	Down	Crucial in metabolic processes, gluconeogenesis, fatty acid synthesis, etc (Leon‐Del‐Rio, 2019)

Up, Upregulation; Down, Downregulation; *P*-value < 0.05; Putative functions were assigned based on literature and database annotations and are provided for contextual interpretation rather than definitive functional claims.

### 3.4 Pathway enrichment analysis

**Figure 3:**
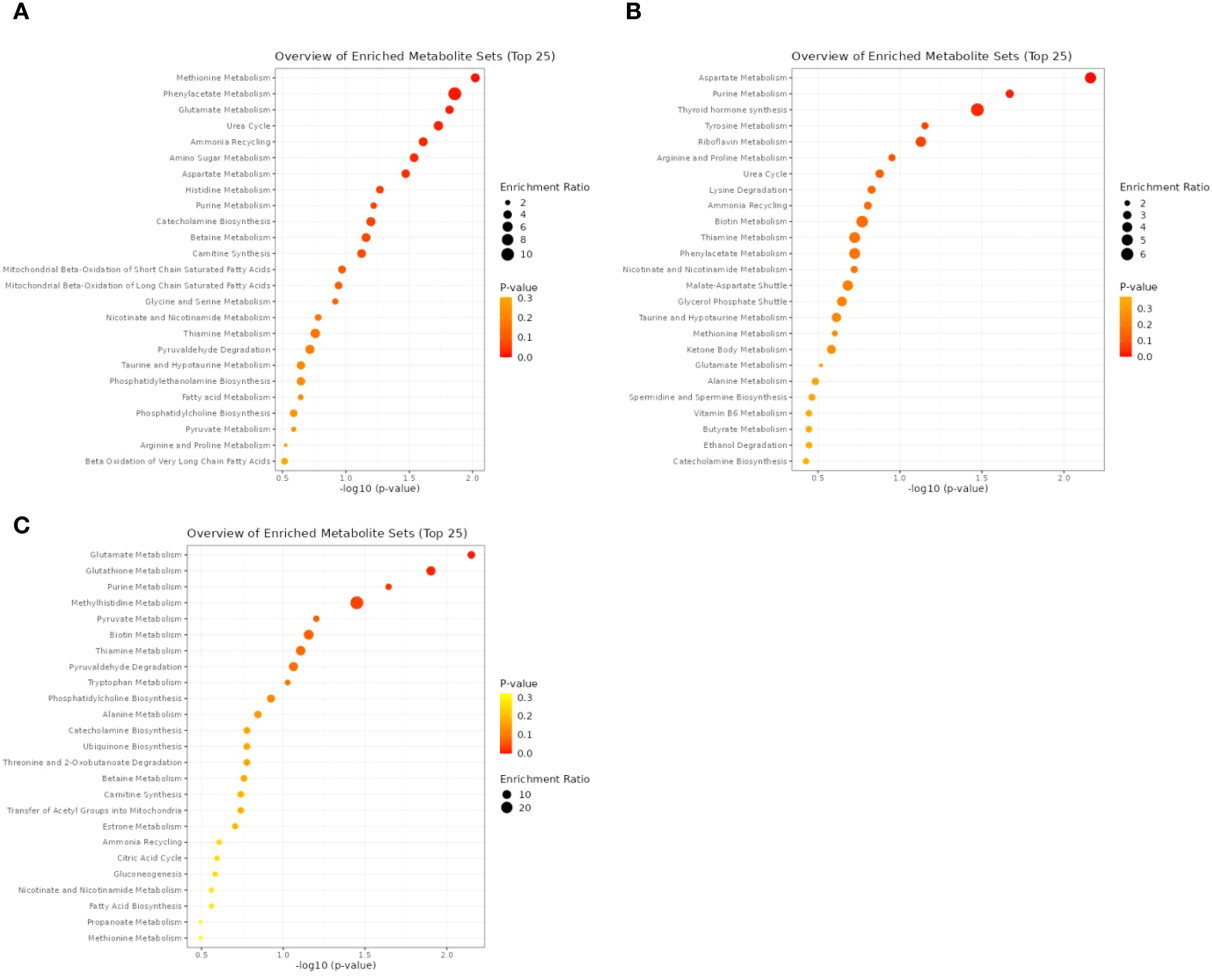
Top 25 kyoto encyclopedia of genes and genomes (KEGG) enriched pathways with -log10 (*P*) > 1.3 (equivalent of *P*  < 0.05) were altered in plasma **(A)**; DRN **(B)**; HYP **(C)** of the metabolome contrast between CON and OLIV groups.

The top 25 KEGG pathways enriched in the plasma, DRN and HYP are shown in **Figures 3A–C**, respectively. Pathways that were significantly enriched in plasma were used as the primary framework for interpretation, after which their corresponding direction of change in the DRN and HYP was evaluated to determine whether systemic alterations were reflected in serotonergic brain regions. The results of the KEGG pathway analysis showed that the OLIV group enriched (*P <* 0.05) a few similar pathways across plasma, DRN and HYP. The result showed that pathways associated with glutamate metabolism was significantly enriched in plasma and HYP and not significantly affected in DRN. The pathway related to aspartate metabolism was significantly enriched in plasma and DRN only, however, pathways related to phenylacetate metabolism and urea cycle were enriched in plasma but not significantly in DRN. Additionally, pathways related to methionine metabolism and ammonia recycling were enriched in plasma but not significantly in DRN and HYP.

### 3.5 Metabolomic pathway topology analysis

**Figure 4:**
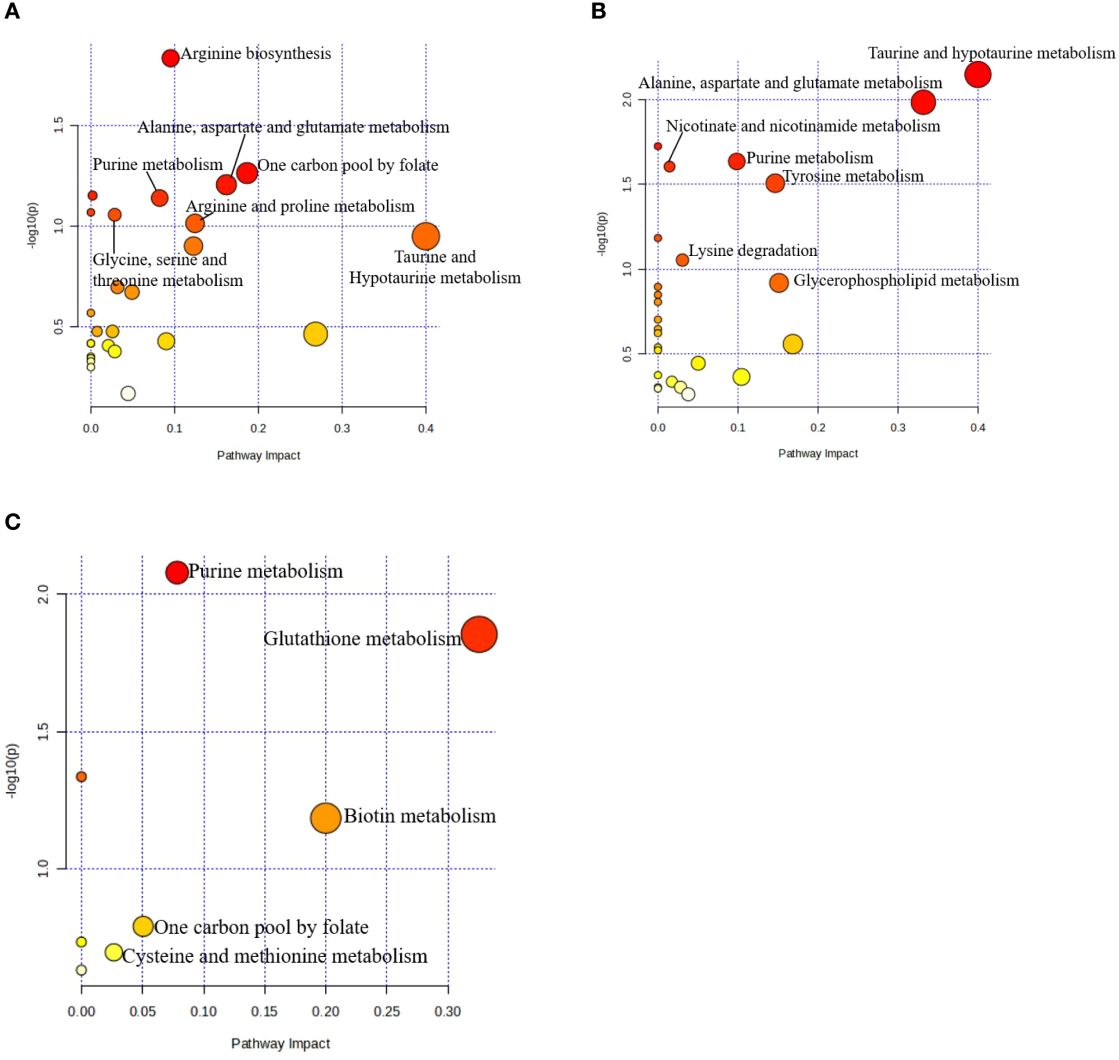
Metabolomic pathway topology analysis of plasma **(A)**; DRN **(B)**; HYP **(C)** of the metabolome contrast between CON and OLIV groups. Pathway impact represents a combination of the centrality and pathway enrichment results as higher impact values represent the relative importance of the pathway. The size of the circle indicates the impact of the pathway while the color represents the significance (the more intense the red color, the lower the p). Pathways with impact > 0.05 and more red color are reported.

Metabolomic pathway topology analysis of plasma, DRN and HYP metabolome contrast between CON and OLIV groups are shown in **Figures 4A–C**. The most important pathways were determined based on impact > 0.05, which are summarized in **Tables 6A–C**. The results of the analysis showed four (4) affected metabolic pathways in the plasma including: arginine biosynthesis; one carbon pool by folate; alanine, aspartate and glutamate metabolism; and purine metabolism. In the DRN, four (4) metabolic pathways including: taurine and hypotaurine metabolism; alanine, aspartate and glutamate metabolism; purine metabolism; and tyrosine metabolism were identified. Two (2) metabolic pathways, including: purine metabolism; and glutathione metabolism were identified in HYP. Moreover, in plasma, DRN and HYP, metabolic pathway related to purine metabolism was commonly altered. Whereas metabolic pathway associated with alanine, aspartate and glutamate metabolism was commonly identified in plasma and DRN only.

**Table 6A:** Metabolomic pathway analysis for metabolites in plasma.

S/N	Pathway name	Total	Hits	Impact
1	Arginine biosynthesis	13	2	0.095
2	One carbon pool by folate	26	2	0.187
3	Alanine, aspartate and glutamate metabolism	28	2	0.162
4	Purine metabolism	67	3	0.082

Pathways with impact > 0.05.

**Table 6B:** Metabolomic pathway analysis for metabolites in DRN.

S/N	Pathway name	Total	Hits	Impact
1	Taurine and hypotaurine metabolism	8	2	0.400
2	Alanine, aspartate and glutamate metabolism	28	3	0.332
3	Purine metabolism	67	4	0.100
4	Tyrosine metabolism	42	3	0.147

Pathways with impact > 0.05.

**Table 6C:** Metabolomic pathway analysis for metabolites in HYP.

S/N	Pathway name	Total	Hits	Impact
1	Purine metabolism	67	3	0.078
2	Glutathione metabolism	28	2	0.325

Pathways with impact > 0.05.

## 4 Discussion

### 4.1 Number of metabolites and principal component analysis

In this study, untargeted metabolomic approaches were employed to evaluate the influence of MUFAs such as olive oil on the composition of metabolites potentially reaching the serotonergic regions of the brain and potential to impact metabolic or behavioral changes in broiler chickens. Significant number of metabolites were detected to pass missing value and blank filter in the plasma, DRN and HYP affirming that the dataset is reliable enough for further data analysis after removing those with a high percentage of missing values across samples and those that show significant signal in blank controls (Schiffman et al., 2019), essentially indicating only the metabolites that are confidently present in the OLIV sample and not just background noise from the analysis process.

The PCA in this study showed distinctly separated clusters in plasma and DRN, which means that the CON and OLIV groups are clearly separated, indicating significant differences between the groups based on the measured variables in the dataset. However, in HYP, cluster separation between CON and OLIV was less pronounced, reflecting greater similarity between treatment groups within this tissue. The overall pattern indicates that metabolic variation was driven more strongly by tissue type than by treatment.

### 4.2 Serotonin concentration

The present study revealed elevated serotonin concentrations in the DRN and HYP of broiler chickens fed dietary olive oil. Increased serotonin in these regions suggests enhanced central serotonergic activity, potentially influenced by olive oil ability to improve membrane lipid composition and reduce oxidative stress factors known to affect tryptophan-serotonin metabolism and neuroendocrine pathways (Sulaiman et al., 2024; Bilal et al., 2021). In poultry, dietary fats, including olive oil, have been associated with changes in neurotransmitterrelated transporters, and olivederived phenolics such as oleuropein have been implicated in modulating appetite and glucose regulation, supporting a neuroactive role for these compounds (Omaliko et al., 2024; Sulaiman et al., 2024). The elevated serotonin observed in the DRN and HYP may therefore reflect heightened serotonergic signaling linked to reduced stress responsiveness and altered feeding behavior. Collectively, these findings indicate that dietary olive oil may modulate the brain-gut axis in broilers, contributing to improved stress coping capacity while influencing growth and feed intake.

### 4.3 Differential abundant metabolites in plasma and brain regions

In the current study, the differential abundant metabolites in both plasma and DRN or HYP suggests the potential migration of essential metabolites to the serotonergic region of the brain. S-succinylcysteine was upregulated in both plasma and DRN. It is a metabolite of protein succination, and an oxidative stress biomarker in the brain, involved in serotonergic signaling to oxidative stress response and neuroprotection (Piroli et al., 2016; Jové et al., 2020). Olive oil supplementation has been shown to enhance antioxidant defenses in broilers, reducing reactive oxygen species (ROS) and lipid peroxidation while increasing superoxide dismutase activity which supports the role of this metabolite as an oxidative stress and neuroprotective marker (Mujahid et al., 2009; Elbaz et al., 2023). A previous study by Jové et al. (2020) reported that S-succinylcysteine formation is often associated with mitigating mitochondrial and oxidative stress in mammalian brains, suggesting a similar role may be obtainable in chickens. Accordingly, Barbalace et al. (2021) reported potentials of extra virgin olive oil to scavenge ROS and reduce lipid peroxidation, thereby protecting neurons from oxidative damage. Thus, S-succinylcysteine, obtained in plasma and DRN in this study suggest that this metabolite may influence the brain by affording antioxidant defense that may help reduce oxidative stress, maximize neuroprotection and cognitive functions. According to this study, sphingomyelin d18:1-C20:0 increased in DRN and decreased in plasma. Sphingomyelin is a major component of myelin sheaths surrounding neuronal axons in the central nervous system and contributes to proper myelination and neural function (Olsen and Færgeman, 2017; Schneider et al., 2019). The high oleic acid content in the olive oil has potentials to supports myelin sheath integrity (Grubić Kezele and Ćurko-Cofek, 2022), by the increased sphingomyelin concentrations in DRN of this study. The consumption of olive oil has been linked with behavioral changes like reduced anxiety and depression in humans, triggered by the modulation of neurotransmitter systems such as serotonin and dopamine (Yubero-Serrano et al., 2019). A study by Hussain et al. (2013) reported that diets rich in MUFAs enhance hippocampal neuronal activity and upregulate proteins involved in synaptic plasticity, which correlated with improved spatial memory and learning in animal models. As such, sphingomyelin concentration in chicken brain increases gradually during development, reaching its highest level in adult chicken brain (Helmy and Morris, 2011; Olsen and Færgeman, 2017), as the increased concentration in the current study may suggest such developmental changes. The downregulation of sphingomyelins in plasma is expected as plasma primarily serves as a transport medium rather than a storage site for structural lipids while the brain retains higher concentrations for cellular membrane integrity. Ala-Val, like other dipeptides, have demonstrated antioxidant properties by potentially protecting cells from oxidative stress (Kopec et al., 2020), exhibit neuroprotective effects by scavenging reactive oxygen species (Küpeli Akkol et al., 2020) that leads to oxidative damage of cells; anti-inflammatory effects by reducing neuroinflammation and maintenance of cognitive functions (Kornicka et al., 2023). Furthermore, olive oil also possesses anti-inflammatory properties that may help alleviate neuroinflammation, a significant contributor to depression and other mood disorders (Lassale et al., 2019). The decreased Ala-Val in the DRN of this present study may be attributed to the decreased concentrations of this metabolite in the plasma leading to their insufficient migration to the DRN to effect significant functions in the chicken.

There are differential abundances of metabolites such as dimethoxyflavone, cytosine and santin in the HYP. As a flavonoid, dimethoxyflavone promotes the activation of inhibitory activity of the butyrylcholinesterase (Tomou et al., 2020), regulating the neurotransmitter activity in the brain. In contrast, olive oil supplementation has been associated to modulate neurotransmitter activity (Yubero-Serrano et al., 2019) influencing behavioral changes. Cytosine modifications play important roles in the chicken brain development and function, as cytosine methylation induces epigenetic modification in the genome regulating gene expression and homeostasis (Li et al., 2015) in chickens. Whereas santin is a trimethoxyflavone, a flavonoid compound that has shown potential to enhance memory, learning, and cognitive capacity (Li et al., 2022). Nevertheless, Sartorius et al. (2012) reported that MUFAs promoted regulation of insulin signaling gene in the brain, leading to beneficial effects on cortical activity, locomotion, and sleep by also enhancing cognitive functions. The downregulation of cytosine and santin metabolites in this study may be attributed to reduced methylation (hypomethylation) of cytosine in CG contexts (Jeong et al., 2021) and inhibition of enzymes that enhances the flavonoid functions (Zhanga et al., 2024).

The differential metabolites, γ-glutamylleucine, epigallocatechin, and dethiobiotin, of olive oil enriched diet were abundant in plasma, DRN and HYP. Leucine is a component of gamma-glutamylleucine (Gamma-Glu-Leu), a dipeptide formed via γ-glutamyltransferase (GGT)-mediated reactions, playing a vital role in brain function and metabolism and serve as a precursor for glutamate, an important neurotransmitter in the brain (Dash et al., 2016). Hence, the enzyme GGT participates in amino acid transfer (Zaragozá, 2020). This enzyme could potentially facilitate the transfer of gamma-glutamylleucine to brains by regulating neurotransmitters in the HYP and improving brain function. Gamma-Glu-Leu may also indirectly support redox balance by influencing glutathione (GSH) dynamics, by modulating gamma-aminobutyric acid (GABA) signaling crucial for mitigating oxidative stress in birds exposed to pathogens or environmental stressors as it is a metabolic product of GGT (Wu et al., 2022). Accordingly, MUFAs support mitochondrial efficiency by enhancing adenosine triphosphate (ATP) synthesis and reducing ROS production. Thus, the metabolite in this study may have tendency to improve mitochondrial function by lowering oxidative stress, indirectly preserving GSH levels that can enhance cognitive activity and mood regulation (Enkler et al., 2023). Epigallocatechin and its metabolites can promote neurite growth in neuronal cells at low concentrations, which is relevant to brain development (Pervin et al., 2019). Also, epigallocatechin has shown protective effects against oxidative damage induced by presence of hydrogen peroxide in brain cells by normalizing hypothalamic-pituitary-adrenal axis activity (Chi et al., 2020). The high concentration of epigallocatechin in HYP may suggest promotion of the cell body to facilitate neurons communication signals with other parts of nervous system to improve stress. This is because MUFAs have shown to enhance cortical activity, promoting locomotion in mice, in contrast to SFAs which decreased brain activity and disrupt sleep patterns in human (Sartorius et al., 2012). This improvement in brain activity and physical behavior suggests MUFAs in this study may positively influence neuronal communication in the broiler chickens. Dethiobiotin is a precursor in the biosynthesis of biotin that plays crucial roles in various metabolic processes such as gluconeogenesis, fatty acid synthesis, amino acid metabolism (Leon‐Del‐Rio, 2019), including those in the brain (Liu et al., 2022).

### 4.4 Enriched metabolic pathways

The pathway enrichment analysis revealed that glutamate metabolism was affected in the plasma, DRN and HYP tissues in the broiler chicks. The metabolism of glutamate occurs primarily in astrocytes, integral in maintaining neurotransmitter pools and facilitating energy metabolism in the hypothalamus, which is synthesized via multiple pathways, including from glucose and amino acid derivatives (Wang et al., 2020). Astrocytes convert synaptic glutamate into glutamine via glutamine synthetase, which neurons then use to regenerate glutamate for synaptic release, this cycle ensures efficient neurotransmitter recycling and prevents excitotoxicity (Tani et al., 2014; Andersen et al., 2021) and supports learning, memory, and adaptive behaviors. Moreover, dysfunctional glutamate-glutamine cycling, could alter GABA synthesis (via glutamate decarboxylase), disrupting inhibitory-excitatory balance (Limón et al., 2021). Consequently, it contributes to anxiety-like behaviors in birds under metabolic stress. Interestingly, diets rich in MUFAs such as olive oil in this study may improve visual cognitive tasks and memory, by enhancing acetylcholine production in HYP via MUFA-driven stabilization of glutamate metabolism by preventing excitotoxicity and supports synaptic health and neuroplasticity (Riviere et al., 2021). Glutamate metabolism is tightly linked to brain energy production, neuron and astrocytes metabolize up to 30% of glutamate via the tricarboxylic acid (TCA) cycle and malate-aspartate shuttle (MAS) to generate ATP, supporting synaptic activity and plasticity to maintain cognitive functions during high metabolic demand (Tani et al., 2014). Comparably, Brose et al. (2014) reported that glutamate/glutamine-derived MUFAs fuel mitochondrial respiration and ATP production, as such, olive oil diets in this study that enriched glutamate metabolism pathway suggests that it may boost energy metabolism in broiler chickens.

Aspartate metabolism was found to be enriched in the plasma and DRN of the broiler chickens. L-Asp conversion to D-aspartate through stereochemical inversion, involving serine racemase and D-aspartate conversion to N-methyl-D-aspartate via methylation (Errico et al., 2018) are major metabolic pathways in aspartate metabolism. It acts as an excitatory neurotransmitter in the central nervous system playing a significant role in brain function and development (Balázs et al., 2012). Aspartate metabolism may influence stress responses directly or via its metabolites, depending on stressor types and brain regions (Czarnecka et al., 2021). This is in line with the study of Silva Figueiredo et al. (2017), reported that MUFAs reduce oxidative stress by lowering lipid peroxidation and enhancing GSH dynamics, preserving mitochondrial function by optimizing MAS, ensuring aspartate played significant role in energy metabolism and neurotransmitter synthesis in DRN, suggesting that aspartate metabolism enriched by olive oil may sustain energy for neurotransmission in the broiler chickens.

### 4.5 Metabolomic topology pathway

The metabolite pathway analysis showed that purine metabolism pathway was altered in plasma, DRN and HYP of the broiler chickens. Purine metabolism is crucial for neuronal differentiation and function, acting as metabolic signals controlling cell growth and proliferation during brain development (Fumagalli et al., 2017), with ATP signaling to generate calcium waves that synchronize cell cycle and migration of neural precursors (Fumagalli et al., 2017). The ATP is essential for maintaining cellular energy levels and sustaining synaptic neurotransmission and interactions between neurons and glial cells (Rimbert et al., 2023). Similarly, MUFAs can be metabolized through mitochondrial β-oxidation, providing an efficient energy source for neurons and glial cells. This process ensures a steady supply of ATP, which is critical for maintaining synaptic activity and cellular homeostasis (Cucchi et al., 2019), thereby supporting cognitive performance, improves alertness and stress resilience. Purine metabolism involves both *de novo* synthesis and salvage pathways (Mizukoshi et al., 2023; Yamada et al., 2024), crucial for nucleotide generation needed for cell growth, tissue repair, which are tightly regulated during brain development.

The alanine, aspartate and glutamate metabolism was altered in plasma and DRN of the broiler chickens. There are interrelationships between alanine, aspartate and glutamate. Alanine and aspartate serve as nitrogen donors for glutamate synthesis with the help of alanine aminotransferase, alanine is converted into pyruvate, transferring nitrogen to glutamate. Nevertheless, aspartate aminotransferase can interconvert to aspartate and glutamate and acting to excite neurotransmitters of serotonin and dopamine in the brain (He and Wu, 2020; Salcedo et al., 2021). Additionally, alanine supports brain energy demands through glucose metabolism. MUFA-rich diets, such as olive oil have been associated with improved cognitive role, reduced irritability, and enhanced physical activity (Barbalace et al., 2021). These effects are attributed to the role of MUFAs in maintaining cellular energy levels and supporting brain health by sustaining cognitive processes like attention, learning, and memory by ensuring ATP availability. These metabolic pathways are crucial for neurotransmitter balance and energy metabolism in the brain. Accordingly, the metabolic pathways commonly observed in plasma and DRN or HYP suggest that similar functional metabolites may improve energy metabolic efficiency, and neurotransmission regulation, which can promote brain function, other neuronal mechanisms and cognitive capacities in broiler chickens.

## 5 Conclusion

In conclusion, this study demonstrates that dietary olive oil derived MUFAs modulate the metabolome of both plasma and serotoninrich brain regions in broiler chickens. The identification of differentially abundant metabolites in the DRN and HYP, together with enriched pathways related to aminoacid metabolism and energy regulation, confirms that olive oil supplementation influences neurochemical processes relevant to brain function. Additionally, the higher serotonin concentrations observed in the DRN and HYP of OLIV fed birds provide further evidence of enhanced central serotonergic activity. At the whole-organism level, these coordinated metabolic and neurochemical shifts indicate that MUFA rich diets can enhance cellular energy production, support neurotransmission, and promote neural resilience, ultimately contributing to improved physiological and behavioral outcomes in broiler chickens.

These findings provide insights into the role of dietary fat in brain metabolism and function, providing potential strategies for enhancing brain health and neurological well-being in poultry production, which can also serve as a model for human study. However, further studies using targeted metabolomics are needed in broiler chickens to validate these metabolite identifications and to establish these compounds or their precursors in olive oil.

## Data Availability

The original contributions presented in the study are included in the article/supplementary material. Further inquiries can be directed to the corresponding author.

## References

[B1] Aklan I. Sayar-Atasoy N. Deng F. Kim H. Yavuz Y. Rysted J. . (2023). Dorsal raphe serotonergic neurons suppress feeding through redundant forebrain circuits. Mol. Metab. 69, 101676. doi: 10.1016/j.molmet.2023.101676. PMID: 36682413 PMC9923194

[B2] Albert P. R. Vahid-Ansari F. Luckhart C. (2014). Serotonin-prefrontal cortical circuitry in anxiety and depression phenotypes: pivotal role of pre-and post-synaptic 5-HT1A receptor expression. Front. Behav. Neurosci. 8, 199. doi: 10.3389/fnbeh.2014.00199. PMID: 24936175 PMC4047678

[B3] Andersen J. V. Markussen K. H. Jakobsen E. Schousboe A. Waagepetersen H. S. Rosenberg P. A. . (2021). Glutamate metabolism and recycling at the excitatory synapse in health and neurodegeneration. Neuropharmacol. 196, 108719. doi: 10.1016/j.neuropharm.2021.108719. PMID: 34273389

[B4] Angeloni C. Malaguti M. Barbalace M. C. Hrelia S. (2017). Bioactivity of olive oil phenols in neuroprotection. Inter. J. Mol. Sci. 18, 2230. doi: 10.3390/ijms18112230. PMID: 29068387 PMC5713200

[B5] Aviagen, W (2022). Ross 708: Broiler management and nutrition specifications. Available online at: https://aviagen.com/assets/Tech_Center/Ross_Broiler/Ross-BroilerNutritionSpecifications2022-EN.pdf (Accessed June 5, 2023).

[B6] Balázs D. Csillag A. Gerber G. (2012). L-aspartate effects on single neurons and interactions with glutamate in striatal slice preparation from chicken brain. Brain Res. 1474, 1–7. doi: 10.1016/j.brainres.2012.07.049. PMID: 22871268

[B7] Barbalace M. C. Zallocco L. Beghelli D. Ronci M. Scortichini S. Digiacomo M. . (2021). Antioxidant and neuroprotective activity of extra virgin olive oil extracts obtained from Quercetano cultivar trees grown in different areas of the Tuscany region (Italy). Antioxidants 10, 421. doi: 10.3390/antiox10030421. PMID: 33801925 PMC8000409

[B8] Bilal R. M. Liu C. Zhao H. Wang Y. Farag M. R. Alagawany M. . (2021). Olive oil: nutritional applications, beneficial health aspects and its prospective application in poultry production. Front. Pharmacol. 12, 723040. doi: 10.3389/fphar.2021.723040. PMID: 34512350 PMC8424077

[B9] Brose S. A. Marquardt A. L. Golovko M. Y. (2014). Fatty acid biosynthesis from glutamate and glutamine is specifically induced in neuronal cells under hypoxia. J. Neurochem. 129, 400–412. doi: 10.1111/jnc.12617. PMID: 24266789 PMC3997620

[B10] Chi X. Ma X. Li Z. Zhang Y. Wang Y. Yuan L. . (2020). Protective effect of epigallocatechin‐3‐gallate in hydrogen peroxide‐induced oxidative damage in chicken lymphocytes. Oxid. Med. Cell. Long. 2020, 7386239. doi: 10.1155/2020/7386239. PMID: 33488931 PMC7790551

[B11] Chianese R. Coccurello R. Viggiano A. Scafuro M. Fiore M. Coppola G. . (2018). Impact of dietary fats on brain functions. Curr. Neuropharmacol. 16, 1059–1085. doi: 10.2174/1570159X15666171017102547. PMID: 29046155 PMC6120115

[B12] Cintra D. E. Ropelle E. R. Moraes J. C. Pauli J. R. Morari J. de Souza C. T. . (2012). Unsaturated fatty acids revert diet-induced hypothalamic inflammation in obesity. PloS One 7, e30571. doi: 10.1371/journal.pone.0030571. PMID: 22279596 PMC3261210

[B13] Cucchi D. Camacho-Muñoz D. Certo M. Pucino V. Nicolaou A. Mauro C. (2019). Fatty acids-from energy substrates to key regulators of cell survival, proliferation and effector function. Cell. Stress 4, 9. doi: 10.15698/cst2020.01.209. PMID: 31922096 PMC6946016

[B14] Czarnecka K. Pilarz A. Rogut A. Maj P. Szymańska J. Olejnik Ł. . (2021). Aspartame—true or false? Narrative review of safety analysis of general use in products. Nutrients 13, 1957. doi: 10.3390/nu13061957. PMID: 34200310 PMC8227014

[B15] Dash P. K. Hergenroeder G. W. Jeter C. B. Choi H. A. Kobori N. Moore A. N. (2016). Traumatic brain injury alters methionine metabolism: implications for pathophysiology. Front. Syst. Neuro. 10, 36. doi: 10.3389/fnsys.2016.00036. PMID: 27199685 PMC4850826

[B16] Elbaz A. M. Zaki E. F. Salama A. A. Badri F. B. Thabet H. A. (2023). Assessing different oil sources efficacy in reducing environmental heat-stress effects via improving performance, digestive enzymes, antioxidant status, and meat quality. Sci. Rep. 13, 20179. doi: 10.1038/s41598-023-47356-6. PMID: 37978201 PMC10656531

[B17] Enkler L. Szentgyörgyi V. Pennauer M. Prescianotto-Baschong C. Riezman I. Wiesyk A. . (2023). Arf1 coordinates fatty acid metabolism and mitochondrial homeostasis. Nat. Cell. Biol. 25, 1157–1172. doi: 10.1038/s41556-023-01180-2. PMID: 37400497 PMC10415182

[B18] Errico F. Nuzzo T. Carella M. Bertolino A. Usiello A. (2018). The emerging role of altered d-aspartate metabolism in schizophrenia: new insights from preclinical models and human studies. Front. Psych. 9, 559. doi: 10.3389/fpsyt.2018.00559. PMID: 30459655 PMC6232865

[B19] Fumagalli M. Lecca D. Abbracchio M. P. Ceruti S. (2017). Pathophysiological role of purines and pyrimidines in neurodevelopment: unveiling new pharmacological approaches to congenital brain diseases. Front. Pharm. 8, 941. doi: 10.3389/fphar.2017.00941. PMID: 29375373 PMC5770749

[B20] Gray T. Fasina Y. O. Harrison S. H. Chang E. M. Chang A. Y. Maldonado-Devincci A. . (2026). Exploring the impact of a high-fat diet on the serotonin signaling in gut-brain axis. Nutr. Neurosci. 29, 40–54. doi: 10.1080/1028415X.2025.2539320. PMID: 40853783

[B21] Grubić Kezele T. Ćurko-Cofek B. (2022). Neuroprotective panel of olive polyphenols: Mechanisms of action, anti-demyelination, and anti-stroke properties. Nutrients 14, 4533. doi: 10.3390/nu14214533. PMID: 36364796 PMC9654510

[B22] Hassamal S. (2023). Chronic stress, neuroinflammation, and depression: an overview of pathophysiological mechanisms and emerging anti-inflammatories. Front. Psychiat. 14, 1130989. doi: 10.3389/fpsyt.2023.1130989. PMID: 37252156 PMC10213648

[B23] He W. Wu G. (2020). Metabolism of amino acids in the brain and their roles in regulating food intake. Amino Acid Nutr. Health: Amino Acids Syst. Funct. Health 1265, 167–185. doi: 10.1007/978-3-030-45328-2_10. PMID: 32761576

[B24] Helmy F. M. Morris A. (2011). A comparative study of the lipid composition of the brain of chicken and rat during myelination. A chromatographic and densitometric analysis. JPC–J. Planar. Chromat.–Modern TLC 24, 325–330. doi: 10.1556/JPC.24.2011.4.10

[B25] Hussain G. Schmitt F. Loeffler J. P. Aguilar J. L. G. D. (2013). Fatting the brain: a brief of recent research. Front. Cellul. Neurosci. 7, 144. doi: 10.3389/fncel.2013.00144. PMID: 24058332 PMC3766822

[B26] Jeong H. Mendizabal I. Berto S. Chatterjee P. Layman T. Usui N. . (2021). Evolution of DNA methylation in the human brain. Nat. Commun. 12, 2021. doi: 10.1038/s41467-021-21917-7. PMID: 33795684 PMC8017017

[B27] Jové M. Pradas I. Mota-Martorell N. Cabré R. Ayala V. Ferrer I. . (2020). Succination of protein thiols in human brain aging. Front. Aging Neurosci. 12, 52. doi: 10.3389/fnagi.2020.00052. PMID: 32210786 PMC7068737

[B28] Kanehisa M. Goto S. (2000). KEGG: Kyoto encyclopedia of genes and genomes. Nucleic Acids Res. 28, 27–30. doi: 10.1093/nar/28.1.27. PMID: 10592173 PMC102409

[B29] Kopec W. Jamroz D. Wiliczkiewicz A. Biazik E. Pudlo A. Korzeniowska M. . (2020). Antioxidative characteristics of chicken breast meat and blood after diet supplementation with carnosine, L-histidine, and β-alanine. Antioxidants 9, 1093. doi: 10.3390/antiox9111093. PMID: 33171823 PMC7695160

[B30] Kornicka A. Balewski Ł. Lahutta M. Kokoszka J. (2023). Umbelliferone and its synthetic derivatives as suitable molecules for the development of agents with biological activities: a review of their pharmacological and therapeutic potential. Pharmaceuticals 16, 1732. doi: 10.3390/ph16121732. PMID: 38139858 PMC10747342

[B31] Küpeli Akkol E. Genç Y. Karpuz B. Sobarzo-Sánchez E. Capasso R. (2020). Coumarins and coumarin-related compounds in pharmacotherapy of cancer. Cancers 12, 1959. doi: 10.3390/cancers12071959. PMID: 32707666 PMC7409047

[B32] Larrieu T. Layé S. (2018). Food for mood: relevance of nutritional omega-3 fatty acids for depression and anxiety. Front. Physiol. 9, 1047. doi: 10.3389/fphys.2018.01047. PMID: 30127751 PMC6087749

[B33] Lassale C. Batty G. D. Baghdadli A. Jacka F. Sánchez-Villegas A. Kivimäki M. . (2019). Healthy dietary indices and risk of depressive outcomes: a systematic review and meta-analysis of observational studies. Molecul.psychiat. 24, 965–986. doi: 10.1038/s41380-018-0237-8. PMID: 30254236 PMC6755986

[B34] Lauretti E. Iuliano L. Praticò D. (2017). Extra‐virgin olive oil ameliorates cognition and neuropathology of the 3xTg mice: role of autophagy. Ann. Clin. Transl. Neurol. 4, 564–574. doi: 10.1002/acn3.431. PMID: 28812046 PMC5553230

[B35] Lenth R. (2024). emmeans: Estimated Marginal Means, aka Least-Squares Means. R package version 1.10.6. Available online at: https://CRAN.R-project.org/package=emmeans.

[B36] Leon‐Del‐Rio A. (2019). Biotin in metabolism, gene expression, and human disease. J. Inherit. Metab. Dis. 42, 647–654. doi: 10.1002/jimd.12073. PMID: 30746739

[B37] Li J. Li R. Wang Y. Hu X. Zhao Y. Li L. . (2015). Genome-wide DNA methylome variation in two genetically distinct chicken lines using MethylC-seq. BMC Genomics 16, 1–13. doi: 10.1186/s12864-015-2098-8. PMID: 26497311 PMC4619007

[B38] Li J. Sun M. Cui X. Li C. (2022). Protective effects of flavonoids against Alzheimer’s disease: Pathological hypothesis, potential targets, and structure–activity relationship. Inter. J. Molecul. Sci. 23, 10020. doi: 10.3390/ijms231710020. PMID: 36077418 PMC9456554

[B39] Limón I. D. Angulo-Cruz I. Sánchez-Abdon L. Patricio-Martínez A. (2021). Disturbance of the glutamate-glutamine cycle, secondary to hepatic damage, compromises memory function. Front. Neurosci. 15, 578922. doi: 10.3389/fnins.2021.578922. PMID: 33584185 PMC7873464

[B40] Liu Y. Liang S. Wang K. Zi X. Zhang R. Wang G. . (2022). Physicochemical, nutritional properties and metabolomics analysis fat deposition mechanism of chahua chicken no. 2 and yao chicken. Genes 13, 1358. doi: 10.3390/genes13081358. PMID: 36011269 PMC9407069

[B41] Martín-Peláez S. Covas M. I. Fito M. Kušar A. Pravst I. (2013). Health effects of olive oil polyphenols: recent advances and possibilities for the use of health claims. Molecul. Nutrit. Food. Res. 57, 760–771. doi: 10.1002/mnfr.201200421. PMID: 23450515

[B42] Mizukoshi T. Yamada S. Sakakibara S. I. (2023). Spatiotemporal regulation of de novo and salvage purine synthesis during brain development. eneuro 10, 1–21. doi: 10.1523/ENEURO.0159-23.2023. PMID: 37770184 PMC10566546

[B43] Mujahid A. Akiba Y. Toyomizu M. (2009). Olive oil-supplemented diet alleviates acute heat stress-induced mitochondrial ROS production in chicken skeletal muscle. Am. J. Physiol. Regul. Intgr. Comp. Physiol. 297, R690–R698. doi: 10.1152/ajpregu.90974.2008. PMID: 19553496

[B44] Nishitani N. Nagayasu K. Asaoka N. Yamashiro M. Andoh C. Nagai Y. . (2019). Manipulation of dorsal raphe serotonergic neurons modulates active coping to inescapable stress and anxiety-related behaviors in mice and rats. Neuropsychopharmacol. 44, 721–732. doi: 10.1038/s41386-018-0254-y. PMID: 30377380 PMC6372597

[B45] Olsen A. S. Færgeman N. J. (2017). Sphingolipids: membrane microdomains in brain development, function and neurological diseases. Open Biol. 7, 170069. doi: 10.1098/rsob.170069. PMID: 28566300 PMC5451547

[B46] Omaliko P. C. Ferket P. R. Ogundare T. E. Apalowo O. O. Enenya I. G. Iwuozo O. C. . (2024). Impact of dietary fat types on expression levels of dopamine and serotonin transporters in the ileum of broiler chickens. Poult. Sci. 103, 104114. doi: 10.1016/j.psj.2024.104114. PMID: 39214056 PMC11402036

[B47] Patrick R. P. Ames B. N. (2015). Vitamin D and the omega‐3 fatty acids control serotonin synthesis and action, part 2: Relevance for ADHD, bipolar disorder, schizophrenia, and impulsive behavior. FASEB J. 29, 2207–2222. doi: 10.1096/fj.14-268342. PMID: 25713056

[B48] Perez-Herrera A. Delgado-Lista J. Torres-Sanchez L. A. Rangel-Zuniga O. A. Camargo A. Moreno-Navarrete J. M. . (2012). The postprandial inflammatory response after ingestion of heated oils in obese persons is reduced by the presence of phenol compounds. Molecular Nutrition & Food Research 56 (3), 510–514. doi: 10.1002/mnfr.201100533 22162245

[B49] Pervin M. Unno K. Takagaki A. Isemura M. Nakamura Y. (2019). Function of green tea catechins in the brain: Epigallocatechin gallate and its metabolites. Inter. J. Molecul. Sci. 20, 3630. doi: 10.3390/ijms20153630. PMID: 31349535 PMC6696481

[B50] Pirman T. Rezar V. Vrecl M. Salobir J. Levart A. (2021). Effect of olive leaves or marigold petal extract on oxidative stress, gut fermentative activity, and mucosa morphology in broiler chickens fed a diet rich in n-3 polyunsaturated fats. J. Poult. Sci. 58, 119–130. doi: 10.2141/jpsa.0200026. PMID: 33927566 PMC8076619

[B51] Piroli G. G. Manuel A. M. Clapper A. C. Walla M. D. Baatz J. E. Palmiter R. D. . (2016). Succination is increased on select proteins in the brainstem of the NADH dehydrogenase (ubiquinone) Fe-S protein 4 (Ndufs4) knockout mouse, a model of Leigh syndrome. Molecul. Cellul. Proteo. 15, 445–461. doi: 10.1074/mcp.M115.051516. PMID: 26450614 PMC4739666

[B52] R Core Team . (2024). R: A Language and Environment for Statistical Computing. (Vienna, Austria: R Foundation for Statistical Computing). Available online at: https://www.R-project.org/.

[B53] Rafei-Tari A. Sadeghi A. Mousavi S. (2021). Inclusion of vegetable oils in diets of broiler chicken raised in hot weather and effects on antioxidant capacity, lipid components in the blood and immune responses. Acta Scientiarum. Animal Sciences 43. doi: 10.4025/actascianimsci.v43I1.50587

[B54] Rimbert S. Moreira J. B. Xapelli S. Lévi S. (2023). Role of purines in brain development, from neuronal proliferation to synaptic refinement. Neuropharmacol. 237, 109640. doi: 10.1016/j.neuropharm.2023.109640. PMID: 37348675

[B55] Riviere A. Georghiades N. Beathard K. Riechman S. (2021). Associations of monounsaturated fats to visual cognitive performance training in older adults. Cur. Dev. Nutri. 5, 920. doi: 10.1093/cdn/nzab049_033

[B56] Rohart F. Gautier B. Singh A. Le Cao K. A. (2017). mixOmics: An R package for 'omics feature selection and multiple data integration. PLoS Computational Biology 13 (11), e1005752. doi: 10.1371/journal.pcbi.1005752 29099853 PMC5687754

[B57] Róman G. C. Jackson R. E. Gadhia R. Róman A. N. Reis J. (2019). Mediterranean diet: The role of long-chain ω-3 fatty acids in fish; polyphenols in fruits, vegetables, cereals, coffee, tea, cacao and wine; probiotics and vitamins in prevention of stroke, age-related cognitive decline, and Alzheimer disease. Revue Neurologique 175 (10), 724–741. doi: 10.1016/j.neurol.2019.08.005 31521398

[B58] Salcedo C. Andersen J. V. Vinten K. T. Pinborg L. H. Waagepetersen H. S. Freude K. K. . (2021). Functional metabolic mapping reveals highly active branched-chain amino acid metabolism in human astrocytes, which is impaired in iPSC-derived astrocytes in Alzheimer’s disease. Front. Aging Neurosci. 13, 736580. doi: 10.3389/fnagi.2021.736580. PMID: 34603012 PMC8484639

[B59] Sartorius T. Ketterer C. Kullmann S. Balzer M. Rotermund C. Binder S. . (2012). Monounsaturated fatty acids prevent the aversive effects of obesity on locomotion, brain activity, and sleep behavior. Diabetes 61, 1669–1679. doi: 10.2337/db11-1521. PMID: 22492529 PMC3379681

[B60] Schiffman C. Petrick L. Perttula K. Yano Y. Carlsson H. Whitehead T. . (2019). Filtering procedures for untargeted LC-MS metabolomics data. BMC Bioinf. 20, 1–10. doi: 10.1186/s12859-019-2871-9. PMID: 31200644 PMC6570933

[B61] Schneider N. Hauser J. Oliveira M. Cazaubon E. Mottaz S. C. O’Neill B. V. . (2019). Sphingomyelin in brain and cognitive development: preliminary data. Eneuro 6, 1–13. doi: 10.1523/ENEURO.0421-18.2019. PMID: 31324675 PMC6709232

[B62] Silva Figueiredo P. Carla Inada A. Marcelino G. Maiara Lopes Cardozo C. De Cássia Freitas K. De Cássia Avellaneda Guimarães R. . (2017). Fatty acids consumption: the role metabolic aspects involved in obesity and its associated disorders. Nutrients 9, 1158. doi: 10.3390/nu9101158. PMID: 29065507 PMC5691774

[B63] Sulaiman U. Vaughan R. Siegel P. Liu D. Gilbert E. R. Cline M. A. (2024). Oleuropein has hypophagic effects in broiler chicks. Front. Physiol. 15, 1409211. doi: 10.3389/fphys.2024.1409211. PMID: 38933363 PMC11199682

[B64] Tan B. L. Norhaizan M. E. (2019). Effect of high-fat diets on oxidative stress, cellular inflammatory response and cognitive function. Nutrients 11, 2579. doi: 10.3390/nu11112579. PMID: 31731503 PMC6893649

[B65] Tani H. Dulla C. G. Farzampour Z. Taylor-Weiner A. Huguenard J. R. Reimer R. J. (2014). A local glutamate-glutamine cycle sustains synaptic excitatory transmitter release. Neuron 81, 888–900. doi: 10.1016/j.neuron.2013.12.026. PMID: 24559677 PMC4001919

[B66] Thaler J. P. Yi C. X. Schur E. A. Guyenet S. J. Hwang B. H. Dietrich M. O. . (2012). Obesity is associated with hypothalamic injury in rodents and humans. J. Clin. Investi. 122, 153–162. doi: 10.1172/JCI59660. PMID: 22201683 PMC3248304

[B67] Tomou E. M. Barda C. Skaltsa H. (2020). Genus Stachys: A review of traditional uses, phytochemistry and bioactivity. Medicines 7, 63. doi: 10.3390/medicines7100063. PMID: 33003416 PMC7601302

[B68] Tonissen S. Tetel V. Fraley G. S. (2022). Transportation stress increases fos immunoreactivity in the paraventricular nucleus, but not in the nucleus of the hippocampal commissure in the pekin duck, Anas platyrhynchos domesticus. Animals 12, 3213. doi: 10.3390/ani12223213. PMID: 36428440 PMC9686473

[B69] Tsugawa H. Cajka T. Kind T. Ma Y. Higgins B. Ikeda K. . (2015). MS-DIAL: data-independent MS/MS deconvolution for comprehensive metabolome analysis. Nature Methods 12, 523–526. doi: 10.1038/nmeth.3393 25938372 PMC4449330

[B70] Valdearcos M. Robblee M. M. Benjamin D. I. Nomura D. K. Xu A. W. Koliwad S. K. (2014). Microglia dictate the impact of saturated fat consumption on hypothalamic inflammation and neuronal function. Cell Rep. 9, 2124–2138. doi: 10.1016/j.celrep.2017.02.008. PMID: 25497089 PMC4617309

[B71] Wang J. Wang F. Mai D. Qu S. (2020). Molecular mechanisms of glutamate toxicity in Parkinson’s disease. Front. Neurosci. 14, 585584. doi: 10.3389/fnins.2020.585584. PMID: 33324150 PMC7725716

[B72] Worley B. Powers R. (2013). Multivariate analysis in metabolomics. Current Metabolomics 1 (1), 92–107. doi: 10.2174/2213235X11301010092 26078916 PMC4465187

[B73] Wu Q. Li J. Zhu J. Sun X. He D. Li J. . (2022). Gamma-glutamyl-leucine levels are causally associated with elevated cardio-metabolic risks. Front. Nutrit. 9, 936220. doi: 10.3389/fnut.2022.936220. PMID: 36505257 PMC9729530

[B74] Yamada S. Mizukoshi T. Sato A. Sakakibara S. I. (2024). Purinosomes and purine metabolism in mammalian neural development: A review. Acta Histochem. Cytochem. 57, 89–100. doi: 10.1267/ahc.24-00027. PMID: 38988694 PMC11231565

[B75] Yubero-Serrano E. M. Lopez-Moreno J. Gomez-Delgado F. Lopez-Miranda J. (2019). Extra virgin olive oil: More than a healthy fat. Europ. J. Clin. Nutrit. 72, 8–17. doi: 10.1038/s41430-018-0304-x. PMID: 30487558

[B76] Zaragozá R. (2020). Transport of amino acids across the blood-brain barrier. Front. Physiol. 11, 973. doi: 10.3389/fphys.2020.00973. PMID: 33071801 PMC7538855

[B77] Zhanga S. Chena W. Maa C. Luoa Y. Donga L. Hua X. . (2024). Role of microbiota-gut-brain axis in neurodegenerative diseases and the potential of microbiome-targeted d ietary strategies: A review of current evidence and future perspective. Food. Sci. Hum. Wellne. 14 (9), 9250215. doi: 10.26599/FSHW.2024.9250215

